# Development and Validation of an Improved HPLC-MS/MS Method for Quantifying Total and Unbound Lenalidomide in Human Plasma

**DOI:** 10.3390/pharmaceutics16101340

**Published:** 2024-10-19

**Authors:** Suhyun Lee, Seungwon Yang, Wang-Seob Shim, Eunseo Song, Seunghoon Han, Sung-Soo Park, Suein Choi, Sung Hwan Joo, Seok Jun Park, Beomjin Shin, Donghyun Kim, Hyeonsu Kim, Yujung Jung, Kyung-Tae Lee, Eun Kyoung Chung

**Affiliations:** 1 Department of Pharmacy, College of Pharmacy, Kyung Hee University, Seoul 02447, Republic of Korea; sh198410@khu.ac.kr (S.L.); syang345@khu.ac.kr (S.Y.); shsonic95@khu.ac.kr (S.H.J.); psjqkr0824@naver.com (S.J.P.); rangers9804@khu.ac.kr (B.S.); waterlion3@khu.ac.kr (D.K.); lucanus2@khu.ac.kr (H.K.); picaso48@naver.com (Y.J.); 2 Department of Regulatory Science, Graduate School, Kyung Hee University, Seoul 02447, Republic of Korea; 3 Institute of Regulatory Innovation through Science, Kyung Hee University, Seoul 02447, Republic of Korea; 4 Kyung Hee Drug Analysis Center, College of Pharmacy, Kyung Hee University, Seoul 02447, Republic of Korea; wsshimm@khu.ac.kr (W.-S.S.); sssk2303@khu.ac.kr (E.S.); 5 Department of Pharmacology, College of Medicine, The Catholic University of Korea, Seoul 06591, Republic of Korea; waystolove@catholic.ac.kr (S.H.); mychloe00@gmail.com (S.C.); 6 Department of Hematology, Hematology Hospital, Seoul St. Mary’s Hospital, The Catholic University of Korea, Seoul 06591, Republic of Korea; imsnake@catholic.ac.kr; 7 Department of Biomedical and Pharmaceutical Sciences, Graduate School, Kyung Hee University, Seoul 02447, Republic of Korea; 8 Department of Pharmacy, Kyung Hee University Hospital at Gangdong, Seoul 05278, Republic of Korea

**Keywords:** lenalidomide, protein binding, quantitative analysis, validation, human plasma, HPLC-MS/MS

## Abstract

Background/Objectives: This study aimed to develop a fully validated HPLC-MS/MS method for quantifying total and unbound lenalidomide concentrations in human plasma. Methods: Unbound concentrations were measured using plasma ultrafiltrate prepared with Amicon^®^ Centrifugal Filters. Lenalidomide and lenalidomide-d5 (internal standard) were extracted from 50 μL of human plasma using liquid–liquid extraction. Chromatography was conducted with a Halo^®^ C18 column using 0.1% formic acid and methanol (20:80, *v*/*v*) as the mobile phase. The mass spectrometer was operated in a positive ion mode with an electrospray ionization interface and multiple reaction monitoring modes. Results: Calibration curves were linear over the range of 5 to 1000 ng/mL (r^2^ > 0.996) for both the total and unbound lenalidomide. For total lenalidomide concentrations, between-run precision (coefficients of variation) and accuracy were 1.70–7.65% and 94.45–101.10%, respectively. For unbound concentrations, inter-day precision and accuracy were 1.98–10.55% and 93.95–98.48%, respectively. Conclusions: We developed a highly reproducible, sensitive, and efficient bioanalytical method using a smaller volume of plasma sample (50 μL) with a relatively short run time (2.5 min). The proposed analytical method was successfully applied to measure total and unbound lenalidomide concentrations at various time points in multiple myeloma patients with renal impairment.

## 1. Introduction

Lenalidomide is an oral immunomodulatory agent with antiangiogenic and antitumor properties [1]. It is primarily used for the treatment of hematologic cancers, including multiple myeloma (MM) [2]. Current practice guidelines recommend lenalidomide in combination with dexamethasone and/or other anticancer agents such as bortezomib as first-line regimens for treatment of MM [3]. Although lenalidomide was initially approved for use with dexamethasone in patients with MM who had received at least one prior therapy, its indication has been substantially expanded [1]. A recent phase 1b/2 study demonstrated the comparable efficacy and safety of lenalidomide in combination with dexamethasone and selinexor for conventional chemotherapy in patients with relapsed or refractory MM, as well as newly diagnosed MM [4]. In addition, the most updated treatment guidelines suggest lenalidomide monotherapy or combination therapy as a maintenance therapy after an autologous hematopoietic stem cell transplant [3]. With these updated guideline recommendations, as well as newer clinical trial findings, use of lenalidomide is expected to increase, particularly with the increasing prevalence of MM due to population ageing [5].

One of the challenges associated with lenalidomide therapy is the substantial variability of lenalidomide in plasma concentrations, potentially leading to concentration-dependent hematologic toxicities such as neutropenia, thrombocytopenia, and anemia [1,6,7]. Considering that lenalidomide is primarily excreted by the kidneys, most of the pharmacokinetic variabilities of lenalidomide might be accounted for by renal function [8,9]. Systemic exposures of lenalidomide could be significantly increased in patients with renal impairment, one of the common complications of MM [10]. Furthermore, severe renal impairments such as end-stage kidney disease might substantially affect the protein binding of drugs, potentially leading to alterations in unbound drug concentrations [11]. Because unbound drug concentrations are responsible for pharmacological and toxicological effects, it is the unbound concentration of a drug that results in an increased risk of toxicity [12,13]. Therefore, for renally impaired patients with advanced MM, the measurement of unbound lenalidomide concentrations is pertinent for individualized precision dosing and therapeutic drug monitoring (TDM), if needed, to optimize treatment outcomes.

With the increasing needs for individualized therapy and the TDM of lenalidomide in patients with MM [14,15,16], particularly those complicated by severe renal impairment, a reliable and validated bioanalytical method is critical to accurately determine unbound lenalidomide concentrations. Several previous studies have reported bioanalytical methods, including high-performance liquid chromatography coupled with tandem mass spectrometry (HPLC-MS/MS) as the preferred method to quantify total lenalidomide concentrations in human plasma [17,18,19,20]. However, those previously developed methods require a relatively large volume of plasma samples (i.e., ≥100 μL) and prolonged run times (i.e., ≥5 min/run) [17,18,19,20]. A high-throughput bioanalytical assay with a smaller volume of samples for analysis, as well as a shorter run time, is imperative to efficiently perform clinical pharmacokinetic studies and TDM in clinical practice. Moreover, none of the previously developed bioanalytical methods were fully validated to quantify unbound lenalidomide in human plasma. A fully validated bioanalytical assay with high efficiency enables rapid, accurate, and precise measurements of unbound lenalidomide concentrations in a large number of human plasma samples, which ultimately support clinical pharmacokinetic studies and TDM for optimal lenalidomide-based therapy for MM. Therefore, the objective of this study was to establish a rapid, efficient, and fully validated bioanalytical method for the accurate and reliable quantification of the total and unbound lenalidomide in human plasma.

## 2. Materials and Methods

### 2.1. Drug Standards and Chemicals

Lenalidomide (purity: 100%) was acquired from Sigma-Aldrich (Steinheim, Germany) and stored in a refrigerator. Lenalidomide-d5 (internal standard (IS), purity: 98.77%, isotopic purity: 95%), was purchased from TRC (Toronto, ON, Canada) and stored frozen at −20 °C. All reagents and solvents were of HPLC analytical grade. A human plasma sample, treated with K2-EDTA, was obtained from BioChemed (Winchester, VA, USA), stored at −80 °C (±10 °C), and used to prepare calibration standards and QC samples.

Stock solutions containing 1 mg/mL of lenalidomide and IS were prepared in 100% dimethyl sulfoxide (DMSO). Working solutions were diluted with 50% methanol in distilled water (*v*/*v*). All stock and working solutions were stored at −20 °C. Quality control (QC) samples were independently prepared in the same manner at concentrations of 15, 300, and 800 ng/mL. Calibration standard samples were prepared from working solutions spiked with blank human plasma to achieve lenalidomide concentrations of 5, 20, 50, 100, 200, 500, and 1000 ng/mL, based on previous lenalidomide pharmacokinetic studies in patients with multiple myeloma [21,22,23,24,25]. Concentrations used for QCs and calibration curves were set the same for both the total and unbound lenalidomide.

### 2.2. Instrumentation and Analytical Conditions

Chromatographic separation was performed using an Agilent 1200 Series HPLC system (Agilent Technologies, Santa Clara, CA, USA) using a Halo^®^ C18 column (Advanced Materials Technology, Wilmington, DE, USA) (100 × 2.1 mm, 2.7 μm) at 40 °C. Mobile phases were tested in an isocratic and a gradient mode with 0.1% formic acid, 0.1% acetic acid, 5 mMol ammonium formate, methanol, and acetonitrile [18,20,21,23,25,26,27,28,29,30,31,32]. The optimized mobile phase consisted of 0.1% formic acid in deionized water and 100% methanol at a 20:80 (*v*/*v*) ratio with the flow rate of 0.2 mL/min [20]. The autosampler temperature was set at 10 °C, and the total chromatographic run time was 2.5 min.

**Figure 1:**
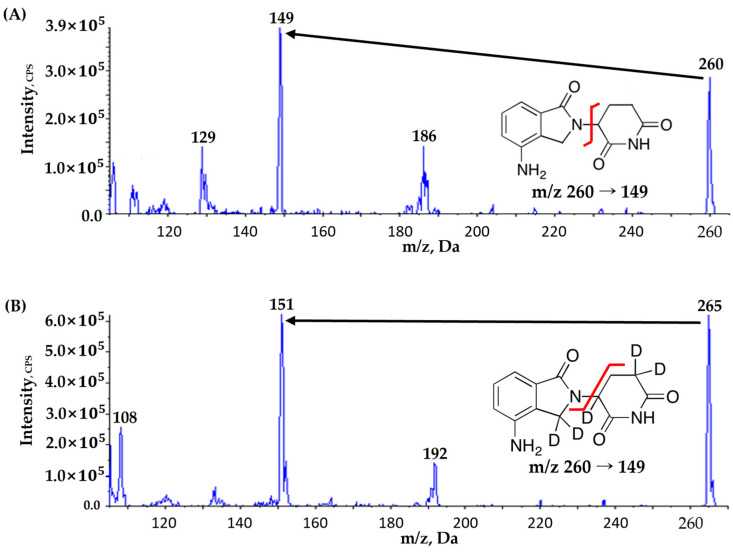
Product ion mass spectra and fragmentation of (**A**) lenalidomide and (**B**) lenalidomide-d5 (IS). Black arrows highlight the primary product ions at *m*/*z* 149 in (**A**) and *m/z* 151 in (**B**), crucial for quantitative analysis in multiple reaction monitoring (MRM) mode. Red lines indicate the specific fragmentation sites leading to the formation of these product ions for lenalidomide and lenalidomide-d5, respectively.

Mass spectrometric detection was performed with the column eluted into the AB SCIEX API 4000 triple quadrupole mass spectrometer (MS) (AB SCIEX, Framingham, MA, USA), operating in a positive ion mode with an electrospray ionization (ESI) and a multiple reaction monitoring (MRM) mode [20]. Data acquisition and processing were managed using Analyst^®^ software version 1.6.2. Optimized MS parameters were as follows (Table 1): ion spray voltage at 5500 V, source temperature at 350 °C, curtain gas flow at 20 psi, and collision gas flow at 7 psi. Lenalidomide and IS (lenalidomide-d5) were detected at transition (*m*/*z*) 260 → 149 and 265 → 151, respectively, based on the fragmentation pattern and signal intensity (Figure 1).

### 2.3. Plasma Sample Preparation

Sample preparation was performed using the liquid–liquid extraction (LLE) technique for quantifying the total and unbound lenalidomide in plasma [17,20,25,29,33]. For the determination of the total lenalidomide concentrations, QC and calibration standards (50 μL each) were freshly prepared by spiking 45 μL of blank human plasma with 5 μL of lenalidomide working solutions of various concentrations. Subsequently, 20 μL of IS working solution (1000 ng/mL) and 1.3 mL of methyl tertiary-butyl ether (MTBE, extraction solvent) were mixed with each of the QC and calibration standards, as previously described [33]. After being shaken at 14,000 rpm for 10 min, the mixtures were centrifuged at 20,000× *g* for 10 min at 4 °C. After the supernatant of the centrifuged sample was transferred to a microtube, it was evaporated under nitrogen gas at 40 °C [20,21]. The dried residue was reconstituted with 1000 μL of 50% methanol and vortexed for 10 min at 20,000× *g* [20,21]. Then, 5 μL of the reconstituted solution was injected into the HPLC–MS/MS system.

**Figure 2:**
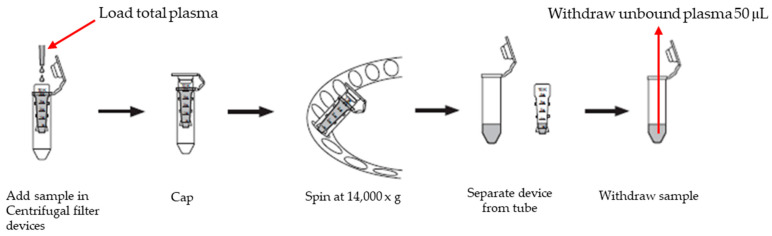
Process of separating unbound lenalidomide from human plasma.

When quantifying unbound lenalidomide in plasma samples, centrifugal filtration was performed to obtain plasma ultrafiltrate by removing bound forms of lenalidomide (Figure 2). Due to the reversible protein binding of lenalidomide in plasma, to maintain a dynamic equilibrium between bound and unbound forms [34], centrifugal filtration was considered a reasonable method to measure the unbound form of lenalidomide. Ultrafiltrate of blank human plasma was prepared with 150 μL of blank human plasma extracted with Amicon^®^ Ultra-0.5 Centrifugal Filter Devices (Sigma-Aldrich; Steinheim, Germany, Figure 2). Unbound QC and calibration standards (50 μL each) were freshly prepared by mixing 5 μL of lenalidomide working solutions of various concentrations with 45 μL of blank human plasma ultrafiltrate.

Afterward, the final unbound sample extracts for injection into the chromatographic column were prepared using the LLE technique as described above, for measurement of total lenalidomide concentrations (Figure 2).

### 2.4. Method Validation

Our newly developed method was validated regarding selectivity, linearity, carryover, precision, accuracy, recovery, matrix effect, and stability, based on the bioanalytical method validation guidelines published by the United States Food and Drug Administration (FDA) and the Ministry of Food and Drug Safety in Korea (MFDS) [35,36].

Selectivity for the quantification of the total lenalidomide in the plasma was determined by analyzing six single-sourced blank human plasma and pooled blank human plasma samples for interferences at the retention times of lenalidomide and IS. For the unbound fraction, blank plasma ultrafiltrate prepared with blank human plasma extracted with Amicon^®^ Ultra-0.5 Centrifugal Filter Devices was used. Acceptance criteria for selectivity were 20% or less of the area or height of the peak response at the lowest limit of quantitation for lenalidomide, and 5% or less of the area or height of the peak response for IS.

To verify linearity (y = ax + b), calibration curves were constructed separately for total and unbound lenalidomide to establish the relationship between lenalidomide concentrations (x) and the peak area ratios (lenalidomide to IS; y) using linear regression weighted with 1/x^2^ over the concentration range from 5 ng/mL (lower limit of quantification, LLOQ) to 1000 ng/mL (upper limit of quantification, ULOQ) [21]. The LLOQ was validated with a signal-to-noise ratio (S/N) of ≥10. Carryover was evaluated by analyzing a double-blank human plasma sample, following the ULOQ standard sample. Acceptance criteria for carryover were the same as those for selectivity.

Precision and accuracy were evaluated at four QC standard concentrations of 5 (LLOQ), 15 (low QC), 300 (medium QC), and 800 (high QC) ng/mL [21,22,23,24]. Within-run precision and accuracy were determined using five replicates for each run within the same day. Between-run precision and accuracy were assessed with five replicates each day for three days. Accuracy and precision were calculated as the percent ratio of the mean predicted to nominal concentrations and the percent coefficient of variation (%CV), respectively. Acceptance criteria for precision and accuracy were within ±15% of %CV and deviations from the nominal concentrations, respectively, except for the LLOQ, at which the acceptance criteria were within ±20%.

Extraction recovery and the matrix effect were evaluated with six different human blank plasma using a pre-extraction spiked matrix, a post-extraction spiked matrix, and pure solutions. Extraction recovery was determined by calculating the peak area ratios of the post- to pre-extraction spiked matrix at three QC concentrations (15, 300, and 800 ng/mL). Matrix effects were assessed by comparing peak areas of post-extraction samples with those of pure solutions.

Stabilities of lenalidomide in stock and working solutions were evaluated in three replicates at low (15 ng/mL) and high QC (800 ng/mL) concentrations, after storing them at room temperature for 3 h (stock solution) and 7 h (working solutions). The stabilities of IS in stock and working solutions were assessed at the concentration of 1000 ng/mL, under the same conditions as lenalidomide. Stability was measured by comparing their peak areas to those of working solutions stored at −20 °C for 7 h. The stability of lenalidomide in plasma was examined in triplicate at three QC concentrations of 15, 300, and 800 ng/mL under the following conditions: freeze–thaw stability after three cycles at −70 °C; short-term stability after storage at room temperature, 4 °C, and −70 °C for 7 h; and extracted sample stability in the autosampler at 10 °C for 30 h. Lenalidomide stability in plasma was measured by comparing the peak areas under each specified condition with those of freshly prepared standards. Acceptance criteria for stability were within ±15% deviation of the mean predicted concentration from its theoretical value.

### 2.5. Statistical Analysis

Descriptive statistics were employed to summarize the validation parameters of the analytical method with arithmetic mean and standard deviation. To assess linearity, linear regression was performed with lenalidomide concentrations as the independent variable (x) and the peak area ratios of lenalidomide to IS as the dependent variable (y), using 1/x^2^ as the weighting factor.

### 2.6. Application of the Method to an Ongoing Pharmacokinetic Study

The newly developed analytical method was applied to three patients with MM receiving 25 mg of lenalidomide orally or equivalent adjusted doses as monotherapy or combination therapy with other agents in those with kidney impairment [37]. The total and unbound plasma concentrations of lenalidomide were measured using the HPLC-MS/MS method developed in this study. After three days of the first cycle of lenalidomide-based chemotherapy, blood samples were collected pre-dose (0 h) and at 2, 4, 8, 12, and 24 h after the oral administration of the study dose. Blood samples were drawn into EDTA-K2 tubes and centrifuged at 3000 rpm for 10 min to obtain plasma. Plasma samples were stored at −70 °C until analysis. This study was approved by the Institutional Review Board of St. Mary’s Hospital in Seoul (IRB No: KC24MISK0219).

## 3. Results and Discussion

### 3.1. Optimization of the HPLC–MS/MS Method

To develop a rapid, accurate, and reliable HPLC-MS/MS method for the quantification of the total and unbound lenalidomide in human plasma, various conditions were examined to optimize bioanalytical performance. The HPLC method was optimized to efficiently separate lenalidomide with sharp peak shape, adequate retention, and free from potential interferences in human plasma. Several reverse-phased columns were examined to optimize peak shape and resolution, including Halo^®^ C18 (100 × 2.1 mm, 2.7 μm), Kinetex^®^ C18 (Phenomenex, Torrance, CA, USA) (100 × 2.1 mm, 2.6 μm), and Cadenza^®^ C18 columns (Imtakt, Kyoto, Japan) (150 × 3.0 mm, 3 μm). While substantial matrix peaks were observed at or near the lenalidomide retention time with the Cadenza^®^ and Kinetex^®^ C18 columns, use of a Halo^®^ C18 column produced the best chromatographic results in terms of peak shape, resolution, and reproducibility without peak tailing or splitting at the column temperature, maintained at 40 °C for consistent chromatographic behavior. Similarly, various solvent compositions were tested as a mobile phase in an isocratic or a gradient mode using 0.1% formic acid, 0.1% acetic acid, 5 mMol ammonium formate, methanol, and acetonitrile. The mobile phase, consisting of 0.1% formic acid in deionized water and 100% methanol (20:80, *v*/*v*) at a flow rate of 0.2 mL/min, resulted in robust separation with good, symmetric peak shapes as well as short and precise retention times of 1.12 and 1.13 min for lenalidomide and IS, respectively. The dead volume time was 0.45 min. Other mobile phase compositions produced matrix peaks with comparable heights to those of lenalidomide peaks, especially at the LLOQ, as well as peak shouldering and a severely curved baseline over the entire run time; thus, they were not selected as the final mobile phase due to the difficulty in the precise and accurate quantitation of the total and unbound lenalidomide in plasma. The total run time was 2.5 min. Considering the dead volume time of 0.45 min, the total run time was optimized to be relatively short (2.5 min), suggesting a rapid bioanalytical assay.

The key parameters for MS were optimized in a positive ion electrospray source at the source temperature of 350 °C to promote desolvation. A protonated molecular ion dominated the full scan of Q1 spectra at *m*/*z* 260 and 265 for lenalidomide and IS, respectively, which served as precursor ions in the MRM mode. The *m*/*z* transitions of 260 to 149 for lenalidomide and 265 to 151 for IS were carefully selected based on fragmentation behavior and signal intensity. Flow rates for curtain gas, collision gas, and ionized gas were optimized to maintain a clean ion path and efficient ion transfer, ultimately enhancing signal intensity for both lenalidomide and IS.

### 3.2. Sample Preparation and IS Selection

To extract the total and unbound lenalidomide from plasma, protein precipitation (PP) and solid-phase extraction (SPE), as well as LLE, were tested as sample preparation methods. Although previous studies utilized SPE and PP with various solvents, including methanol [18,20,21,26,27], this study showed substantial matrix peaks at the lenalidomide retention time, suggesting poor chromatographic separation. In this study, the best chromatographic separation was achieved with LLE for both total and unbound lenalidomide using MTBE, which has a lower polarity than the ethyl acetate used as an extraction solvent in previous studies [18,20,21,26,27].

In contrast to previous studies using 100 to 300 μL of plasma for lenalidomide quantitation [18,20,21,26,27], our study utilized a much smaller volume of plasma sample (i.e., 50 μL), making our newly developed method a more efficient assay for the high-throughput analysis required for clinical pharmacokinetic studies and TDM. In addition, the use of a smaller plasma volume sufficiently removed potentially interfering endogenous substances including proteins and lipids, thus substantially improving matrix effects [18,20,21,26,27]. To further reduce the influence of the plasma matrix, deuterium substituents of lenalidomide were employed as IS rather than structural analogs of lenalidomide such as pomalidomide.

### 3.3. Method Validation

To our knowledge, this study is the first study fully validating the bioanalytical method to measure unbound plasma concentrations of lenalidomide. For quantitating the total lenalidomide in human plasma, our analytical assay showed adequate validity, satisfying the national and international guidance on bioanalytical method validation [35,36]. Specifically, the selectivity, precision, accuracy, and stability were comparable between our method and previously developed methods, with negligible carryover [20,25,26,27,28,29,30,31,32,38]. The linearity range was reasonable, from 5 to 1000 ng/mL, compared to previous studies (0.078–259.26 ng/mL to 65–30,000 ng/mL) [19,31]. Our method extracted analytes sufficiently without substantial interferences, consistent with previous methods [20,25,26,27,28,29,30,31,32,38]. Detailed validation test results for our newly developed bioanalytical assay are provided below.

#### 3.3.1. Selectivity and Sensitivity (LLOQ)

**Figure 3:**
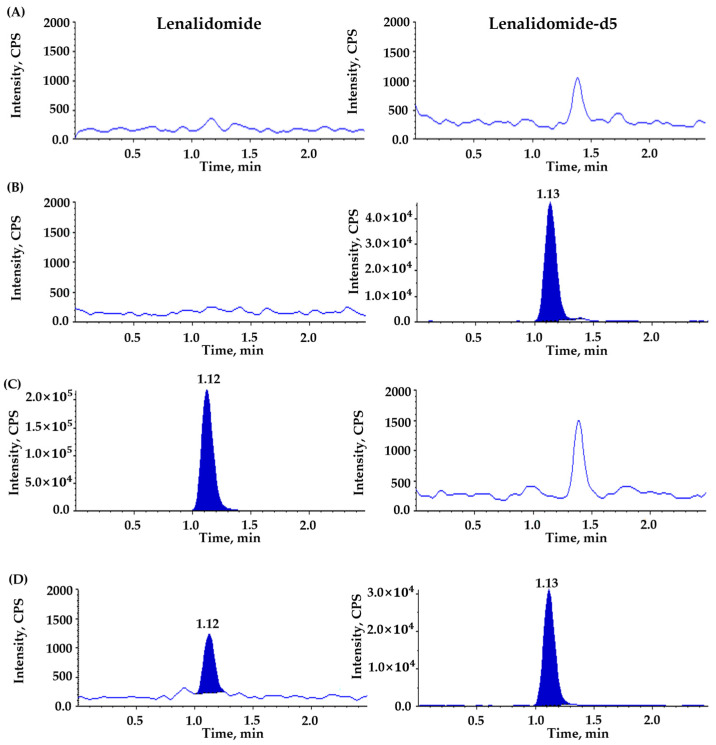
Representative chromatograms for total lenalidomide in plasma using (**A**) double blank sample, (**B**) blank sample spiked with lenalidomide-d5 as internal standard (IS, 1000 ng/mL), (**C**) blank sample spiked with lenalidomide (upper limit of quantification, 1000 ng/mL), (**D**) blank sample spiked with lenalidomide (lower limit of quantification, 5 ng/mL) and lenalidomide-d5 (IS, 1000 ng/mL). Panels on the left side are for lenalidomide, and those on the right side for IS.

**Figure 4:**
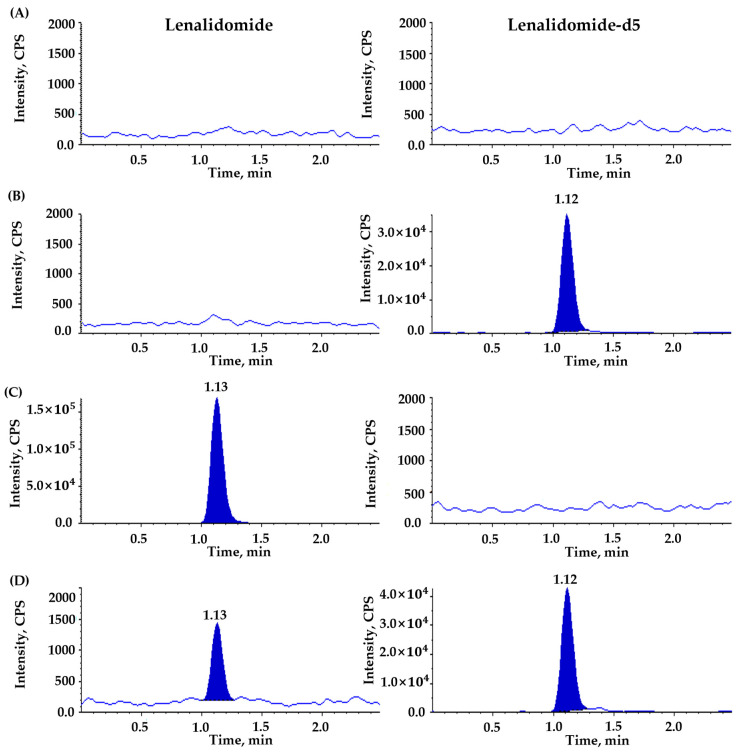
Representative chromatograms for unbound lenalidomide in post-ultrafiltration plasma using (**A**) double blank sample, (**B**) blank sample spiked with lenalidomide-d5 as internal standard (IS, 1000 ng/mL), (**C**) blank sample spiked with lenalidomide (upper limit of quantification, 1000 ng/mL), (**D**) blank sample spiked with lenalidomide (lower limit of quantification, 5 ng/mL) and lenalidomide-d5 (IS, 1000 ng/mL). Panels on the left side are for lenalidomide, and those on the right side for IS.

No significant interferences were observed at the retention time of lenalidomide and IS, demonstrating adequate selectivity and specificity of the newly developed bioanalytical assay to quantitate the total and unbound lenalidomide in plasma (Figure 3 and Figure 4). A ghost but not significant peak was detected at 1.38 min in the chromatographic baseline of IS for blank plasma samples without IS [Figure 3A and Figure 4C]. However, this ghost peak disappeared after ultrafiltration for the measurement of unbound lenalidomide in plasma [Figure 4A,C]. The LLOQ for the total and unbound lenalidomide was the same at 5 ng/mL with S/N ratios ≥ 10 for both, suggesting the adequate sensitivity of the analytical method to quantify the total and unbound lenalidomide in plasma.

#### 3.3.2. Linearity

Based on the total and unbound calibration curves for lenalidomide over the concentration range of 5 to 1000 ng/mL, the linearity of the newly developed analytical method was validated for both the total and unbound lenalidomide in human plasma. The coefficients of determination and the correlation coefficients were ≥0.9990 and ≥0.9995, respectively, for total lenalidomide calibration curves based on a 1/x^2^ weighted linear regression. For unbound lenalidomide, the coefficients of determination and the correlation coefficients were ≥0.9966 and ≥0.9983, respectively. Equations for the total and unbound lenalidomide calibration curves with mean ± standard deviation (SD) slope and intercept were as follows:

- Total lenalidomide: y = 0.00557 (±0.00014) x + 0.00121 (±0.000180)
- Unbound lenalidomide: y = 0.00558 (±0.00009) x + 0.00556 (±0.00157)

#### 3.3.3. Precision and Accuracy

Table 2 summarizes within- and between-run precision and accuracy, expressed as %CV and % ratio of the mean predicted concentration to the nominal concentration. Intra- and inter-run precisions were ≤3.29% and ≤7.65%, respectively, for total lenalidomide measurements; for the unbound lenalidomide assay, within- and between-day precisions were ≤5.01% and ≤10.55%, respectively. Intra- and inter-day accuracies ranged from 93.00 to 105.86% and from 94.45 to 101.10%, respectively, for the determination of the total lenalidomide in the plasma; for unbound lenalidomide, within- and between-run accuracies ranged from 92.14 to 99.42% and from 93.95 to 98.48%, respectively. Overall, our newly developed bioanalytical assays for the total and unbound lenalidomide in human plasma satisfied the pre-defined acceptance criteria of precision and accuracy according to the guidance of the MFDS and the FDA for bioanalytical method validation.

#### 3.3.4. Recovery, Matrix Effect, and Carryover

After the sample pretreatment using LLE to determine total lenalidomide concentrations in human plasma, the mean extraction recovery at three QC concentrations ranged from 49.35 to 58.43% (Table 3). For unbound lenalidomide, the mean extraction recovery after plasma ultrafiltration followed by sample preparation with LLE ranged from 42.83 to 46.75% (Table 3). The additional ultrafiltration step required for plasma samples might account for the relatively lower extraction recovery for unbound lenalidomide assays compared to total lenalidomide analysis. Nevertheless, the estimated CVs (%) were ≤3.82%, indicating the consistent and efficient extraction of lenalidomide from plasma using a smaller plasma volume of 50 μL for both the total and unbound assays (Table 3). Overall, the extraction recovery results suggest high reproducibility of our sample preparation methods, with sufficient recovery of analytes for the quantification of both the total and unbound lenalidomide in human plasma.

The mean matrix effects for the total and unbound lenalidomide assay at three QC concentrations ranged from 99.69 to 106.82% and from 95.81 to 100.04%, respectively (Table 3). The mean matrix effects for the total and unbound IS were 99.15% and 97.33%, respectively (Table 3). Our matrix effect results showed no significant ion enhancement or suppression effects. The matrix effect showed low variability, with CVs of 1.62% for the total lenalidomide and 2.16% for unbound lenalidomide (Table 3), indicating that it remained stable and consistent across different plasma samples. Therefore, our newly developed analytical method was free of considerable matrix effects.

In the carryover study, negligible carryover was observed with the extracted blank sample injected after analyzing the ULOQ standard. Our carryover experiment validated the reliability of sequential sample analysis in our developed bioanalytical assay to quantitate the total and unbound lenalidomide in human plasma.

#### 3.3.5. Stability

The stability (mean ± SD) of lenalidomide in stock solutions was 106.75 ± 5.37% at 15 ng/mL and 102.39 ± 1.56% at 800 ng/mL; for IS, its stability in stock solutions was 105.99 ± 4.60%. The stability of the working solutions was 100.04 ± 5.00% at 15 ng/mL and 102.14 ± 1.28% at 800 ng/mL for lenalidomide, and 100.36 ± 2.80% for IS. The total and unbound lenalidomide were considered stable in plasma samples, with the mean percentage stability ranging from 94.00 to 101.49% for total lenalidomide and from 91.98 to 99.06% for unbound lenalidomide (Table 4). Overall, our stability study results suggest analyte integrity in plasma samples as well as stock and working solutions during routine laboratory handling and analysis without the substantial degradation of lenalidomide under various tested conditions.

### 3.4. Method Applications and Future Directions

**Figure 5:**
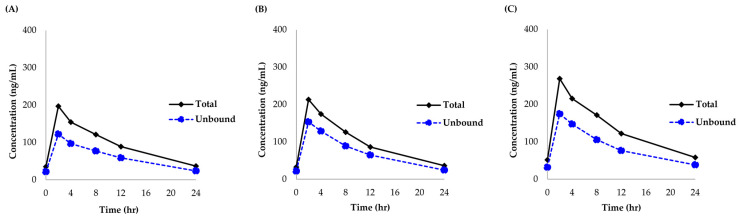
Total and unbound lenalidomide concentrations in plasma from three patients after three days of the first cycle containing oral lenalidomide 25 mg or equivalent dose for renally impaired patients. (**A**–**C**) represent the total and unbound plasma concentrations of lenalidomide in each patient.

The newly developed and validated analytical method was successfully applied to quantitate lenalidomide concentrations in 36 plasma samples from a pharmacokinetic study of lenalidomide in renally impaired patients with MM (*n* = 3). Figure 5 presents the concentration-time profiles of the total and unbound lenalidomide for these patients. All patients reached the peak plasma concentrations of total and unbound lenalidomide within approximately 4 h of oral administration. Plasma concentrations of both the total and unbound lenalidomide in all three patients were measured within the calibration range of our newly developed and validated bioanalytical method (total plasma concentration range of lenalidomide: 32.30 to 268.26 ng/mL, unbound plasma concentration range of lenalidomide: 21.53 to 174.80 mg/L). The median (min to max) unbound fraction was estimated to be 65.5% (61.0 to 75.0%).

Considering its high efficiency, accuracy, and reproducibility, our newly developed and fully validated HPLC-MS/MS assay may be an appropriate high-throughput assay to determine unbound as well as total lenalidomide concentrations from a large number of samples, as in clinical studies and routine TDM in clinical practice. Previous studies and the FDA label information regarding the pharmacokinetic profiles of lenalidomide may adequately justify our newly developed method, including the calibration curve range [2]. In addition to clinical research settings, our newly developed analytical method can be potentially applied to clinical practice for monitoring lenalidomide concentrations in patients, particularly for special populations such as patients with severe renal impairment, where the accurate quantification of unbound concentrations is pertinent for safe and effective drug therapy. Therefore, this study might contribute to enhancing our knowledge regarding lenalidomide pharmacokinetics, especially with respect to protein binding, as well as optimizing lenalidomide therapy through individualized precision dosing based on unbound plasma concentrations, particularly in special populations such as those with severe renal impairment.

## 4. Conclusions

In conclusion, this study reported the development of an efficient, accurate, and reliable HPLC-MS/MS method to measure the total and unbound concentrations of lenalidomide in human plasma. Human plasma was pre-treated with ultrafiltration to determine the unbound lenalidomide concentrations. Our newly developed bioanalytical method, using a smaller volume of plasma sample (i.e., 50 μL) with a relatively short run time of 2.5 min, was fully validated with respect to selectivity, linearity, carryover, precision, accuracy, recovery, matrix effect, and stability. Overall, our highly efficient, accurate, and novel HPLC-MS/MS assay might be successfully used as a high-throughput assay for quantitating lenalidomide from a large number of human plasma samples, making it applicable to routine TDM for lenalidomide in clinical practice, as well as to clinical pharmacokinetic studies for the individualized precision therapy of lenalidomide.

## Figures and Tables

**Table 1:** Mass spectrometer conditions for lenalidomide and lenalidomide-d5 (IS).

Compounds	Ion Transition (*m*/*z*)	DP (V)	EP (V)	CE (V)	CXP (V)	RT (min)	Source Temperature (°C)	Curtain Gas (psi)	GS1 (L/hr)	GS2 (L/hr)
Lenalidomide	260 → 149	71.0	10.0	21.0	24.0	1.12	350	20	40	50
Lenalidomide -d5	265 → 151	96.0	10.0	21.0	26.0	1.13				

DP: declustering potential; EP: entrance potential; CE: collision energy; CXP: cell exit potential; RT: retention time; GS: ionized gas.

**Table 2:** Within- and between-run precision and accuracy for quantification of total and unbound lenalidomide in human plasma.

Nominal Concentration (ng/mL)	Total Lenalidomide			Unbound Lenalidomide		
	Predicted Concentration (Mean ± SD) (ng/mL)	Precision (CV, %) ^a^	Accuracy (%) ^b^	Predicted Concentration (Mean ± SD) (ng/mL)	Precision (CV, %) ^a^	Accuracy (%) ^b^
	Within-run accuracy and precision (*n* = 5 sample replicates)					
5	5.29 ± 0.17	3.29	105.86	4.61 ± 0.19	4.06	92.14
15	15.20 ± 0.31	2.04	101.36	14.39 ± 0.72	5.01	95.91
300	293.75 ± 1.89	0.65	97.92	298.26 ± 5.51	1.85	99.42
800	744.01 + 12.09	1.62	93.00	776.20 ± 5.63	0.73	97.02
	Between-run accuracy and precision (*n* = 3 runs with 5 replicates/run)					
5	5.06 ± 0.39	7.65	101.10	4.92 ± 0.52	10.55	98.48
15	14.97 ± 0.48	3.22	99.80	14.09 ± 0.82	5.80	93.95
300	297.42 ± 5.04	1.70	99.14	292.15 ± 6.59	2.26	97.38
800	755.59 ± 15.45	2.05	94.45	763.95 ± 15.15	1.98	95.49

^a^ CV (%) = (standard deviation of the predicted concentrations/nominal concentration) × 100. ^b^ Accuracy (%) = (predicted concentration/nominal concentration) × 100.

**Table 3:** Extraction recovery and matrix effect of the total and unbound fraction of lenalidomide and lenalidomide-d5 (internal standard, IS) in human plasma (*n* = 6 different human blank plasma).

Nominal Concentration (ng/mL)	Extraction Recovery (Mean ± SD, %)		Matrix Effect (Mean ± SD, %)	
	Total	Unbound Fraction	Total	Unbound Fraction
	Lenalidomide			
15	49.35 ± 1.65	45.26 ± 1.18	106.82 ± 3.08	100.04 ± 1.50
300	55.23 ± 0.86	42.83 ± 0.84	103.15 ± 0.67	98.16 ± 3.39
800	58.43 ± 0.95	46.75 ± 0.66	99.69 ± 0.89	95.81 ± 0.92
	Lenalidomide-d5 (internal standard, IS)			
1000	35.48 ± 1.16	27.73 ± 1.06	99.15 ± 2.72	97.33 ± 1.94

**Table 4:** Stability for total and unbound lenalidomide in human plasma ^a^.

Stability Condition	Nominal Concentration (ng/mL)					
	15		300		800	
	Total Lenalidomide	Unbound Lenalidomide	Total Lenalidomide	Unbound Lenalidomide	Total Lenalidomide	Unbound Lenalidomide
Freeze–thaw (three cycles)	97.97 ± 2.56	91.98 ± 6.81	99.49 ± 0.81	97.66 ± 1.29	97.08 ± 1.07	94.64 ± 0.55
Room temperature (7 h)	94.00 ± 5.11	92.04 ± 6.13	99.96 ± 1.51	98.45 ± 2.16	97.55 ± 1.36	96.28 ± 0.52
4 °C (7 h)	94.41 ± 0.71	96.93 ± 9.15	100.36 ± 2.34	97.34 ± 2.71	96.21 ± 2.61	96.40 ± 1.46
−70 °C (7 h)	96.41 ± 3.41	97.33 ± 4.96	100.55 ± 1.09	99.06 ± 0.95	97.14 ± 0.89	95.54 ± 2.87
Autosampler, 10 °C (30 h)	98.34 ± 2.86	94.49 ± 7.00	101.49 ± 0.68	98.53 ± 0.350	96.60 ± 1.80	93.38 ± 1.42

^a^ Data are presented as % mean ± standard deviation (SD).

## Data Availability

The data presented in this study may be available upon request.
